# New insights into the mortality risk from nasopharyngeal cancer in the national cancer institute formaldehyde worker cohort study

**DOI:** 10.1186/s12995-019-0224-2

**Published:** 2019-02-20

**Authors:** Matthias Möhner, Yimeng Liu, Gary M. Marsh

**Affiliations:** 1 Federal Institute for Occupational Safety and Health, Berlin, Germany; 2 Center for Occupational Biostatistics and Epidemiology and Department of Biostatistics, 7120 Graduate School of Public Health, University of Pittsburgh, 130 DeSoto Street, Pittsburgh, PA 15261 USA

**Keywords:** Formaldehyde, Nasopharyngeal cancer, Cohort mortality study, Occupational health, National Cancer Institute, Re-analyses

## Abstract

**Background:**

Indications were found that a diagnostic bias could have contributed to the National Cancer Institute’s (NCI) suggestion of a persistent increased mortality risk for nasopharyngeal cancer (NPC).

**Methods:**

NCI provided the cohort data updated through 2004. We computed local county rate-based standardized mortality ratios (SMRs) for NPC and all other entities of the pharynx for two time periods. Moreover, SMRs were calculated for pharyngeal cancer in relation to study site by cumulative exposure to formaldehyde (FA).

**Results:**

Overall, our results corroborate the indications of a diagnostic bias by strong but contrary temporal trends for NPC and pharynx, not specified. Moreover, it was shown that mortality risks were increased in the Wallingford cohort for all pharyngeal cancer combined and for pharyngeal cancer excluding NPC. In contrast, no increased risks for these categories were found in the nine other study sites combined.

**Conclusions:**

Our re-analysis provided little or no evidence to support NCI’s suggestion of a persistent association between FA exposure and mortality from NPC.

In October 2009, a working group of the International Agency for Research on Cancer (IARC) unanimously reaffirmed the classification of formaldehyde as Group 1, based on sufficient evidence in humans of nasopharyngeal cancer (NPC), although a group of different working group members concluded in March of the same year that exposure to formaldehyde is unlikely responsible for the increased risks of NPC in woodworkers reported in most case–control studies and in a pooled reanalysis of cohort studies [1, 2]. The reason for this somewhat contradictory assessment - workers in the plywood industry are one of the occupational groups most exposed to formaldehyde [3] - is probably the National Cancer Institute (NCI) formaldehyde worker cohort study, the extended follow-up until 2004 of which had just been completed, even if the first publication of these data was dedicated to lymphohematopoietic malignancies [4]. This NCI cohort is the largest cohort of formaldehyde-exposed industrial workers and thus of particular interest for the evaluation of carcinogenicity. However, the IARC’s conclusion takes only into account the update of follow-up until 1994 [5], which later turned out to be incomplete by comparing the number of deaths with those detected by the next matching with the National Death Index [4]. An updated analysis of nasopharyngeal cancer was not published until 2013, after it became apparent that the former results [5] were biased by the incompleteness of the follow-up [6, 7].

## Re-analysis of the NCI formaldehyde cohort

A recent re-analysis of the NCI formaldehyde cohort showed that the observed overall increased risk for NPC was driven by results for only one study site (Plant 1: Wallingford), and the results for the other nine study sites (Plants 2–9) were unremarkable [8]. The striking concentration of NPC cases in the Wallingford site had already been noted in the first analysis of the NCI-cohort [9, 10], but was not taken into account by NCI investigators in subsequent analyses based on extended follow-ups. In addition, an independent historical cohort and nested case-control study of workers from the Wallingford site found that the large NPC risk was strongly associated with external employment in the ferrous and non-ferrous metal industries of the local area (in particular, silversmithing) that entailed possible exposures to several suspected risk factors for upper respiratory system cancer (e.g., sulfuric acid mists, mineral acid, metal dusts and heat) [11].

## Extended re-analysis of the NCI formaldehyde cohort

Indications were also found in a second independent reanalysis that diagnostic bias could have contributed to the elevated risk estimators for NPC, and it was suggested to carry out an expanded analysis of the NCI formaldehyde cohort to explore these findings further [12]. The analysis is based on the updated 2004 NCI formaldehyde cohort study data from NCI, the same data base as previously used by Marsh and colleagues [8]. Moreover, the external comparison of mortality from pharyngeal cancer is also based on local mortality rates as before, which helps to adjust for geographic, cultural and economic factors possibly related to pharyngeal cancer mortality over time [8]. The recoding of one case, mentioned by Lucas [13] was not taken into account, because it is based on an autopsy or hospital report and not on the death certificate. However, a change of the database from mortality data to cancer registry data would only be permissible if a corresponding record-linkage were made for all study subjects.

The current re-analysis was extended to all subcategories of pharyngeal cancer (International Classification of Diseases, 8th revision (ICD8): 146–149). In order to recognize changes over calendar time, the time period of the initial analysis (1934–1979) was compared with that of the two extension periods for the cohort’s follow-up (1980–2004). Moreover, we compared results between the Wallingford site with those from all other study sites combined.

Table 1 shows that 21 of 50 (42%) observed cases of deaths from pharyngeal cancer were coded as pharynx, unspecified (ICD8: 149). This is exactly in line with the corresponding expected number of cases, calculated based on local mortality rates (42.4%). However, a large difference in the percentage of observed deaths coded as pharynx, unspecified, exists between the two time periods (one death out of 15 (6.7%) versus 20 out of 35 (57.1%), respectively), whereas, this is not observed in the corresponding expected number of deaths (38.5% versus 43.8%, respectively). A comparison of the SMRs for single entities with regard to their distribution over the two time periods reveals a significant decrease in SMR for NPC (ICD8: 147) from 3.23 to 1.11. The test for heterogeneity [14] yields χ^2^ = 4.40 (*p* < 0.05). In contrast, for pharynx, unspecified (ICD8: 149), a strong increase of the SMR from 0.23 to 1.52 was observed (χ^2^ = 3.54 (*p* = 0.06). These results give clear indications of a diagnostic bias or even death certificate coding errors.

**Table 1:** Distribution of pharyngeal cancer cases in the NCI-cohort by sub-localization and period of follow-up

ICD8	1934–1979			1980–2004			1934–2004		
	Obs	Exp	SMR (95%CI)	Obs	Exp	SMR (95%CI)	Obs	Exp	SMR (95%CI)
146 (Oropharynx)	5	2.96	1.69 (0.55–3.95)	8	8.74	0.92 (0.40–1.80)	13	11.70	1.11 (0.59–1.90)
147 (Nasopharynx)	7	2.17	3.23 (1.30–6.65)	4	3.61	1.11 (0.30–2.84)	11	5.78	1.90 (0.95–3.40)
148 (Hypopharynx)	2	1.85	1.08 (0.13–3.91)	3	4.57	0.66 (0.14–1.92)	5	6.42	0.78 (0.25–1.82)
149 (Pharynx, unspecified)	1	4.37	0.23 (0.01–1.28)	20	13.19	1.52 (0.93–2.34)	21	17.56	1.20 (0.74–1.83)
146–9 (Pharynx)	15	11.34	1.32 (0.74–2.18)	35	30.12	1.16 (0.81–1.62)	50	41.46	1.21 (0.90–1.59)

Our analysis by NCI study site showed that mortality risks were increased in the Wallingford cohort for all pharyngeal cancer combined and for pharyngeal cancer excluding NPC (Table 2). In contrast, no increased risks for these categories were found in the nine other study sites combined, clearly demonstrating the significant differences in pharyngeal cancer risk between the Wallingford site and all other study sites. The test for homogeneity [14] supports this observation (χ^2^ = 8.37; *p* < 0.01) as did the statistically significant (plant group x formaldehyde exposure) interaction term computed in the recent re-analysis by Marsh and colleagues [8].

**Table 2:** Distribution of pharyngeal cancer cases in the NCI-cohort by sub-localization and study site (1934–2004)

ICD8	Study site	Obs	Exp	SMR	95% CI
147 (NPC)	Wallingford	6	1.08	5.57	2.04–12.1
146,148,149	Wallingford	11	6.58	1.67	0.83–2.99
146–149	Wallingford	17	7.66	2.22	1.29–3.55
147 (NPC)	All other plants	5	4.70	1.06	0.35–2.48
146,148,149	All other plants	28	29.09	0.96	0.64–1.39
146–149	All other plants	33	33.79	0.98	0.67–1.37
147 (NPC)	Total	11	5.78	1.90	0.95–3.40
146,148,149	Total	39	35.67	1.09	0.78–1.49
146–149	Total	50	41.46	1.21	0.90–1.59

The comparison of exposure categories with regard to cumulative exposure gives no indication of a possible exposure-risk relationship either for the original or for the extended follow-up period (Table 3). A further comparison by plant group shows that the mortality increase for pharyngeal cancer in the Wallingford cohort is virtually the same in all exposure categories with the exception of the non-exposed group (Table 4). These findings support the hypothesis that external occupational exposures in the Wallingford cohort may be causative for the observed excess in pharyngeal cancer mortality.

**Table 3:** Distribution of pharyngeal cancer cases in the NCI-cohort by cumulative exposure to formaldehyde and period of follow-up

Exposure (ppm-yr)	1934–1979			1980–2004			1934–2004		
	Obs	Exp	SMR (95%CI)	Obs	Exp	SMR (95%CI)	Obs	Exp	SMR (95%CI)
0	2	1.06	1.89 (0.23–6.83)	3	2.43	1.24 (0.25–3.61)	5	3.49	1.43 (0.47–3.35)
(0, 0.5]	8	3.59	2.23 (0.96–4.39)	15	12.25	1.22 (0.69–2.02)	23	15.84	1.45 (0.92–2.18)
(0.5, 5.5]	3	4.13	0.73 (0.15–2.12)	9	9.94	0.90 (0.41–1.72)	12	14.07	0.85 (0.44–1.49)
> 5.5	2	2.56	0.78 (0.09–2.82)	8	5.50	1.46 (0.63–2.87)	10	8.06	1.24 (0.59–2.28)
Total	15	11.34	1.32 (0.74–2.18)	35	30.12	1.16 (0.81–1.62)	50	41.46	1.21 (0.90–1.59)

**Table 4:** Distribution of pharyngeal cancer cases in the NCI-cohort by cumulative exposure to formaldehyde and study site (1934–2004)

Exposure (ppm-yr)	Plant 1 (Wallingford)			Plant 2–10 (all other plants)		
	Obs	Exp	SMR (95%CI)	Obs	Exp	SMR (95%CI)
0	0	0.77	0.00 (0.00–4.82)	5	2.72	1.84 (0.60–4.29)
(0, 0.5]	6	2.21	2.71 (1.00–5.90)	17	13.63	1.25 (0.73–2.00)
(0.5, 5.5]	6	2.85	2.10 (0.77–4.57)	6	11.21	0.54 (0.20–1.17)
> 5.5	5	1.83	2.73 (0.89–6.38)	5	6.23	0.80 (0.26–1.87)
Total	17	7.66	2.22 (1.29–3.55)	33	33.79	0.98 (0.67–1.37)

## Conclusions

The analyses presented here provide no any evidence for a relationship between exposure to formaldehyde and pharyngeal cancer mortality in Plants 2–10. The likelihood of a diagnostic or death certificate coding bias with respect to pharyngeal cancer appears be high, especially in the Wallingford cohort. Furthermore, the new findings support the hypothesis that occupational exposures external to the Wallingford cohort may be responsible for the excess of pharyngeal cancer in the Wallingford cohort. Our findings are consistent with other recent study results and reviews [15–20] and they do not support the NCI’s and IARC’s suggested causal association between formaldehyde and the risk of NPC.
